# Is the Increasing Incidence of Thyroid Cancer in the Nordic Countries Caused by Use of Mobile Phones?

**DOI:** 10.3390/ijerph17239129

**Published:** 2020-12-07

**Authors:** Michael Carlberg, Tarmo Koppel, Lena K. Hedendahl, Lennart Hardell

**Affiliations:** 1 The Environment and Cancer Research Foundation, Studievägen 35, SE 702 17 Örebro, Sweden; lenahedendahl@telia.com (L.K.H.); lennart.hardell@environmentandcancer.com (L.H.); 2 School of Business and Governance, Tallinn University of Technology, SOC353 Ehitajate Tee 5, 19086 Tallinn, Estonia; tarmo.koppel@taltech.ee

**Keywords:** mobile phone, cordless phone, thyroid cancer, Swedish Cancer Register, NORDCAN, radiofrequency electromagnetic fields, RF-EMF, ionizing radiation, incidence, Nordic countries

## Abstract

The International Agency for Research on Cancer (IARC) at the World Health Organization (WHO) categorized in 2011 radiofrequency (RF) as a possible human carcinogen, Group 2B. During use of the handheld wireless phone, especially the smartphone, the thyroid gland is a target organ. During the 21st century, the incidence of thyroid cancer is increasing in many countries. We used the Swedish Cancer Register to study trends from 1970 to 2017. During that time period, the incidence increased statistically significantly in women with average annual percentage change (AAPC) +2.13%, 95% confidence interval (CI) +1.43, +2.83%. The increase was especially pronounced during 2010–2017 with annual percentage change (APC) +9.65%, 95% CI +6.68, +12.71%. In men, AAPC increased during 1970–2017 with +1.49%, 95% CI +0.71, +2.28%. Highest increase was found for the time period 2001–2017 with APC +5.26%, 95% CI +4.05, +6.49%. Similar results were found for all Nordic countries based on NORDCAN 1970–2016 with APC +5.83%, 95% CI +4.56, +7.12 in women from 2006 to 2016 and APC + 5.48%, 95% CI +3.92, +7.06% in men from 2005 to 2016. According to the Swedish Cancer Register, the increasing incidence was similar for tumors ≤4 cm as for tumors >4 cm, indicating that the increase cannot be explained by overdiagnosis. These results are in agreement with recent results on increased thyroid cancer risk associated with the use of mobile phones. We postulate that RF radiation is a causative factor for the increasing thyroid cancer incidence.

## 1. Introduction

One established risk factor for thyroid cancer, especially of the papillary type, is ionizing radiation [1]. The first reports of an increased risk were published in the late 1940s and early 1950s [2,3]. Since then, studies have associated thyroid cancer with diagnostic x-ray investigation [2], external radiotherapy [4], and nuclear fallout after the use of A-bombs in Hiroshima and Nagasaki [5] as well as after the Chernobyl and Fukushima disasters [6]. In Belarus, fallout of radioiodine after the Chernobyl accident has been associated with increased incidence of thyroid cancer in children and adolescents [7]. No clear excess to thyroid cancer related to caesium-137 deposition was found in Sweden [8].

Thyroid cancer is more common in women than in men. Hormonal and reproductive factors may explain that difference [9,10].

During the last two decades, a striking increase in the incidence has been reported in the Nordic countries [11]. Use of computed tomography (CT) and positron emission tomography–computed tomography (PET-CT) for diagnostic procedures may have contributed to the increased incidence, but does not seem to explain the whole increase [12,13].

The thyroid is exposed to radiofrequency (RF) radiation during use of mobile and cordless (DECT) phones [14]. This is especially the situation for smartphones that have been increasingly used since the early 2000s. In our previous study, we postulated that exposure to RF-radiation might be a causative factor for the increasing incidence [11].

A case-control study on mobile phone use suggested an increased risk for thyroid cancer [15]. The same material was used to study genotype–environment interaction between single nucleotide polymorphism (SNPs) and mobile phone use [16]. The study showed that mobile phone use increased the risk for thyroid cancer when genetic variants were present within some genes. It was concluded that pathways related to DNA repair may be involved in the increased risk for thyroid cancer associated with mobile phone use. The increased risk was seen regardless of tumor size, ≤10 mm *versus* >10 mm, or latency, ≤13 years *versus* >13 years [16].

The U.S. National Toxicology Program (NTP) results on the toxicology and carcinogenicity of RF radiation in rats and mice showed no increased thyroid cancer incidence in mice [17]. A statistically significant increased incidence of C cell hyperplasia was found in the two years of GSM exposed groups (1.5, 3, and 6 W/kg, respectively) of female rats [18]. In males, a statistically non-significant increased incidence was observed in the 1.5 W/kg exposure group. C cell hyperplasia is a precursor to familial medullary thyroid cancer in humans and may also be a precursor to other types of thyroid cancer [19].

We studied the incidence of thyroid cancer using the Swedish Cancer Register and NORDCAN for the Nordic countries. Our previous published results [11] were thus updated. Since no personal identification was used, no ethical permission was needed.

## 2. Materials and Methods 

### 2.1. Study Design

The National Board of Health and Welfare administers the Swedish Cancer Register. It has yearly updates and was started in 1958. The tumor diagnosis is based on clinical examination, histology/cytology, surgery, and/or autopsy. Additionally, laboratory investigations, CT, and MRI are used for diagnosis.

The ICD-7 code 194 is used in the Swedish Cancer Register for thyroid cancer. We studied the aged-adjusted incidence per 100,000 person years according to the world population for the time period 1970–2017. The database was updated until 2018, but the last year was excluded due to a delay in the reporting of cases leading to an underestimation of number of tumors for that year according to the Swedish Cancer Register (note in the online database). The data are available online (https://sdb.socialstyrelsen.se/if_can/val.aspx). 

NORDCAN was used for all Nordic countries (Sweden, Denmark, Finland, Norway, and Iceland) to study thyroid cancer incidence (ICD-10 code C73) for the time period 1970–2016 (latest update). Thereby age-adjustment was made according to the world population. The data are available online (http://www-dep.iarc.fr/NORDCAN/english/frame.asp).

### 2.2. Statistical Methods

Trends in age-standardized incidence of thyroid cancer by fitting a model of 0–5 joinpoints settings in default mode was analyzed using the 

National Cancer Institute (NCI) Joinpoint Regression Analysis program, version 4.8.0.1 [20]. Annual percentage changes (APC) and 95% confidence intervals (CI) were calculated for each linear segment when joinpoints were detected. For the whole time period, average annual percentage changes (AAPC) were calculated using the average of the APCs weighted by the length of the segment. To calculate APC and AAPC, the data were log-transformed prior to analysis. Thus, it was not possible to perform joinpoint regression analysis when there were years with no cases during the time period. 

## 3. Results

### 3.1. The Swedish Cancer Register

For the whole study period of 1970–2017, the incidence increased statistically significantly in women with AAPC +2.13% (95% CI +1.43, +2.83%), Table 1. Three joinpoints were detected, 1979, 1999, and 2010 with especially high APC for the last period 2010–2017; APC +9.65% (95% CI +6.68, +12.71%). AAPC was statistically significantly increased in all age groups except for the oldest age of 80+ years. It is to be noted that AAPC also increased statistically significantly among the youngest persons of 0–19 years with +1.69% (95% CI +0.88, +2.51%).

**Figure 1:**
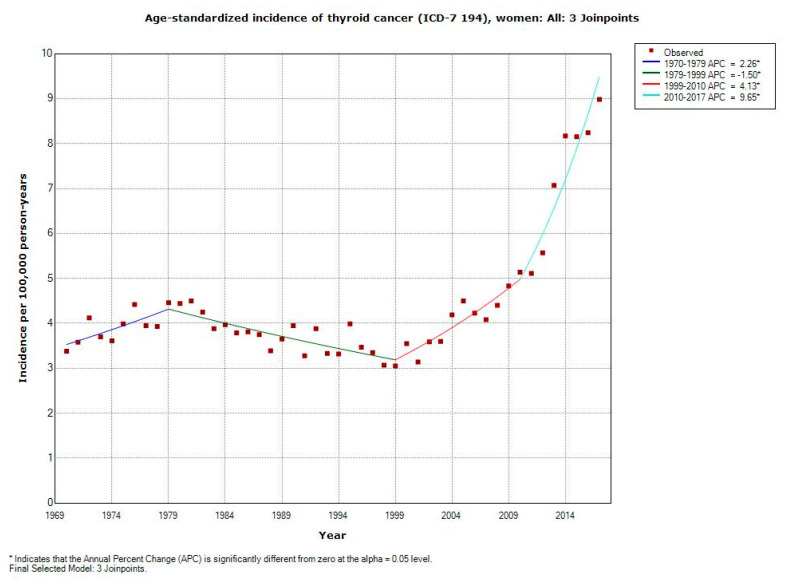
Joinpoint regression analysis of age-standardized incidence of thyroid cancer for women, all ages 1970–2017. Incidence per 100,000 inhabitants for ICD-7 code 194 according to the Swedish Cancer Register (https://sdb.socialstyrelsen.se/if_can/val.aspx).

Figure 1 shows the increasing incidence in women from 1999. Note, especially from 2010, a steep increasing curve.

According to Table 2, the increasing incidence of thyroid cancer was less dramatic in men during 1970–2017 with AAPC +1.49% (95% CI +0.71, +2.28%). AAPC increased statistically significantly in all age groups except for 80+ years. Due to few persons in the youngest age group 0–19 years (*n* = 98), AAPC could not be calculated. The highest increasing APC was found in men aged 20–39 years for the time period 2001–2017; +7.80%, (95% CI +4.17, 11.54%).

**Figure 2:**
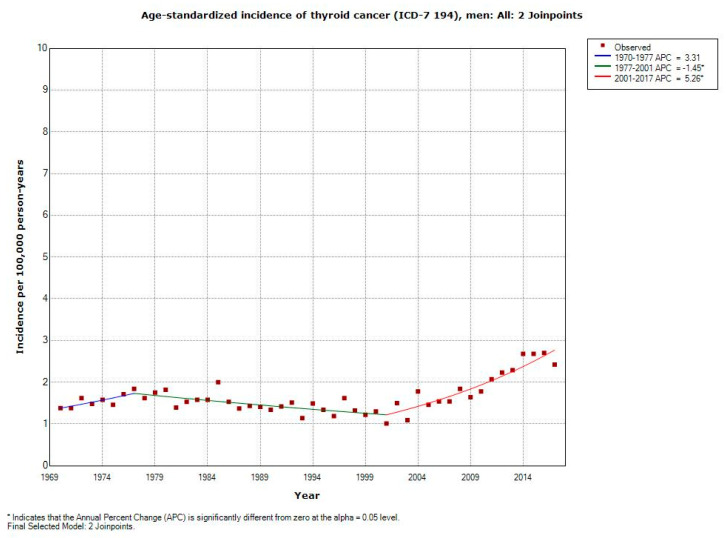
Joinpoint regression analysis of age-standardized incidence of thyroid cancer for men, all ages 1970–2017. Incidence per 100,000 inhabitants for ICD-7 code 194 according to the Swedish Cancer Register (https://sdb.socialstyrelsen.se/if_can/val.aspx).

The age-standardized incidence of thyroid cancer (ICD-194) per 100,000 using joinpoint regression analysis is shown in Figure 2. In men, an increasing incidence is shown from 2001.

### 3.2. NORDCAN

In women, based on NORDCAN, the incidence of thyroid cancer increased statistically significantly during 1970–2016 with AAPC +2.18% (95% CI +1.73, +2.64%), Table 3. 

**Figure 3:**
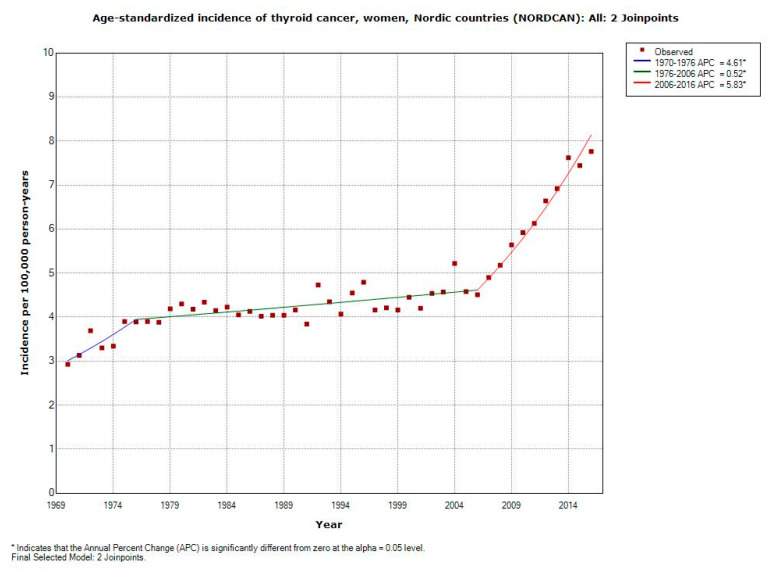
Joinpoint regression analysis of age-standardized incidence of thyroid cancer for women, all ages 1970–2016. Incidence per 100,000 inhabitants for ICD-10 code C73 in the Nordic countries according to NORDCAN (https://www-dep.iarc.fr/NORDCAN/english/frame.asp).

This was based on 36,050 female cancer cases. Two joinpoints were found: 1976 and 2006. During 2006 to 2016, APC increased statistically significantly with +5.83% (95% CI +4.56, +7.12%). These results are also shown in Figure 3. 

**Figure 4:**
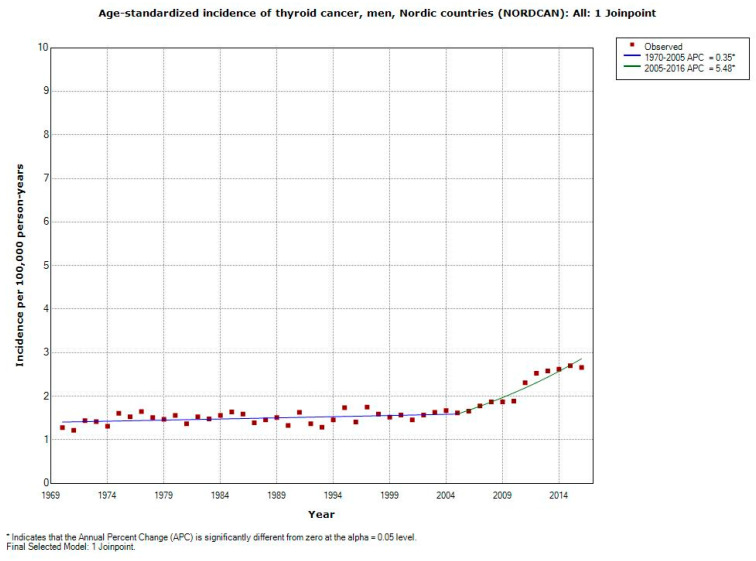
Joinpoint regression analysis of age-standardized incidence of thyroid cancer for men, all ages 1970–2016. Incidence per 100,000 inhabitants for ICD-10 code C73 in the Nordic countries according to NORDCAN (https://www-dep.iarc.fr/NORDCAN/english/frame.asp).

The incidence increased statistically significantly in men during 1970–2016 with AAPC +1.55% (95% CI +1.15, +1.96%), Table 3. Highest increase was found during more recent years with joinpoint 2005; APC +5.48% (95% CI +3.92, +7.06%), see also Figure 4.

## 4. Discussion

The main result in this study was increasing thyroid cancer incidence in Sweden during the study period 1970–2017, especially during the more recent years in both men and women. The increase was even higher than during 1970–2013, as presented in our previous publication [11]. Thus, AAPC in women was now +2.13% compared with +1.19% during our previous study period. The corresponding results in men were +1.49% and +0.77%, respectively. In men, the AAPC increase was now statistically significant.

Increasing statistically significant AAPC was found in all age groups for both genders except those aged 80+ years and in men 0–19 years. These results were based on lower numbers and are similar to those in our previous publication [11]. In both men and women, increasing APC was found from early 2000. Thus, since 2002, thyroid cancer incidence increased in women yearly more than +7% in the age groups 20–39 and 40–59 years. From 2003, APC increased with +6% in the age group 60–79 years. It is noteworthy that for all women, the yearly increase was almost +10% during 2010–2017. A similar pattern was found in men with APC about +5% to almost +8% in the three age groups 20–79 years since the beginning of the 21st century.

According to NORDCAN, the thyroid cancer incidences increased statistically significantly from 2006 in women and from 2005 in men based on a fairly large number of cancer cases. The increase was comparable in both women and men during these time periods at +5.83% *versus* +5.48%.

Since this is a register-based study, the results must be interpreted with caution. The results were given for age groups and were gender-specific. However, these results do definitely indicate an etiologic impact of an exogenous cancer-causing factor with increasing exposure over time. In our previous publication [11], we made a comprehensive discussion of different risk factors. One risk factor might be pollution [21]. Another good candidate is no doubt the use of wireless phones, especially the handheld smartphone that due to the antenna position gives RF exposure to the thyroid gland. This organ is one of the highest exposed aside from the brain during the use of smartphones [14]. These phones have been increasingly common since the early 21st century and are now the only types that are marketed. The first generations of mobile phones were introduced in the 1980s as well as the cordless desktop phone (DECT). Additionally, these handheld phones give off RF radiation to the head and neck region.

A fairly short latency period has been found for ionizing radiation induced thyroid cancer with increasing risk beginning 5–10 years after radiation [22]. Thus, the sharp current increase in thyroid cancer incidence may be radiation induced, most likely RF radiation from the handheld phone. It is noteworthy that Luo et al. [16] also found increased risk in the shortest latency period of ≤13 years.

Increasing thyroid cancer incidence has been seen worldwide during the last 20 years, and is expected to be the fourth most common cancer by 2030 [23]. The rising trend has been reported in different continents with different health systems and ethnicities [24].

So called overdiagnosis has been suggested to explain the increasing thyroid cancer incidence [12] due to better access to health care and screening. However, screening for thyroid cancer is not performed in the Nordic countries and there are no social or demographic differences for health access. Diagnostic patterns might be a contributing factor but do not explain the increasing incidence, especially during recent years [25]. This is supported by the recent results also showing increased risk from RF radiation for tumors ≤10 mm [16]. Interestingly, according to the Swedish Cancer Register, there is a similar incidence increase during 2005–2018 for tumors ≤4 cm (T1-T2) as for tumors >4 cm (T3-T4), or with invasive growth into surrounding tissue. The percentage between these groups was about the same over the years, indicating that the increasing incidence is not due to overdiagnosis [26].

Ionizing radiation is an established risk factor. Increasing use of x-ray investigations for diagnostic procedures may be contributing to the increased incidence, especially for the radio-sensitive papillary type [27]. Chest CT and whole-body trauma CT are increasingly used, but does not explain the pattern of increasing incidence of thyroid cancer; for discussion, see Carlberg et al. [11]. Diet has been suggested to be of etiologic importance for thyroid cancer. In a prospective study, intake of iodine-rich foods and goitrogens were indicated to influence the risk [28]. However, there have been no known sudden changes of the food habits in the Nordic countries that can explain our findings.

## 5. Conclusions

Thyroid cancer incidence has been steeply increasing in Sweden and all Nordic countries during the 21st century. Use of the handheld mobile phone is increasing, in particular, the smartphone gives high RF radiation exposure to the thyroid gland. It is postulated that this might be a causative factor for the increasing incidence supported by human epidemiology that has shown an association between mobile phone use and thyroid cancer.

## Figures and Tables

**Table 1:** Joinpoint regression analysis of thyroid cancer incidence in women in the Swedish Cancer Register 1970–2017. ICD-7 code 194 (https://sdb.socialstyrelsen.se/if_can/val.aspx). APC = Annual Percentage Change (APC 1 = time from 1970 to first joinpoint; APC 2 = time from first joinpoint to 2017 or to second joinpoint; APC 3 = time from second joinpoint to 2017 or to third joinpoint; APC 4 = time from third joinpoint to 2017); AAPC = Average Annual Percentage Change.

ICD-7	Joinpoint Location	APC 1 (95% CI)	APC 2 (95% CI)	APC 3 (95% CI)	APC 4 (95% CI)	AAPC (95% CI)
194						
All women (*n* = 13,020)	1979; 1999; 2010	+2.26 (+0.36, +4.20)	−1.50 (−2.10, −0.90)	+4.13 (+2.48, +5.81)	+9.65 (+6.68, +12.71)	+2.13 (+1.43, +2.83)
0–19 years (*n* = 318)	No joinpoint detected	-	-	-	-	+1.69 (+0.88, +2.51)
20–39 years (*n* = 2935)	2002	+0.53 (+0.07, +0.99)	+7.16 (+5.64, +8.70)	-	-	+2.60 (+2.05, +3.15)
40–59 years (*n* = 4223)	2002	−0.95 (−1.60, −0.29)	+7.39 (+5.20, +9.63)	--	-	+1.64 (+0.85, +2.43)
60–79 years (*n* = 4166)	1974; 2003	+9.50 (−2.44, +22.91)	−2.11 (−2.70, −1.52)	+6.00 (+4.20, +7.83)	-	+1.20 (+0.06, +2.35)
80+ years (*n* = 1378)	1979; 1996	+2.40 (−1.75, +6.73)	−4.57 (−6.22, −2.90)	+0.52 (−0.63, +1.69)	-	−1.00 (−2.08, +0.10)

**Table 2:** Joinpoint regression analysis of thyroid cancer incidence in men in the Swedish Cancer Register 1970–2017. ICD-7 code 194 (https://sdb.socialstyrelsen.se/if_can/val.aspx). APC = Annual Percentage Change (APC 1 = time from 1970 to first joinpoint; APC 2 = time from first joinpoint to 2017 or to second joinpoint; APC 3 = time from second joinpoint to 2017); AAPC = Average Annual Percentage Change.

ICD-7	Joinpoint Location	APC 1 (95% CI)	APC 2 (95% CI)	APC 3 (95% CI)	AAPC (95% CI)
194					
All men (*n* = 5047)	1977; 2001	+3.31 (−0.78, +7.56)	−1.45 (−2.11, −0.79)	+5.26 (+4.05, +6.49)	+1.49 (+0.71, +2.28)
0–19 years (*n* = 98)	-	-	-	-	-
20–39 years (*n* = 800)	2001	−0.67 (−1.92, +0.60)	+7.80 (+4.17, +11.54)	-	+2.13 (+0.72, +3.57)
40–59 years (*n* = 1508)	2003	−0.58 (-1.25, +0.09)	+5.54 (+2.99, +8.16)	-	+1.21 (+0.36, +2.06)
60–79 years (*n* = 2184)	1980; 2001	+2.69 (−0.10, +5.56)	−2.52 (−3.46, −1.57)	+4.77 (+3.36, +6.20)	+1.02 (+0.17, +1.87)
80+ years (*n* = 457)	No joinpoint detected	-	-	-	−1.45 (−2.64, −0.24)

**Table 3:** Joinpoint regression analysis of thyroid cancer incidence in women and men in the Nordic countries according to NORDCAN 1970–2016, ICD-10 code C73 (https://www-dep.iarc.fr/NORDCAN/english/frame.asp). APC = Annual Percentage Change (APC 1 = time from 1970 to first joinpoint; APC 2 = time from first joinpoint to 2016 or to second joinpoint; APC 3 = time from second joinpoint to 2016); AAPC = Average Annual Percentage Change.

ICD-10	Joinpoint Location	APC 1 (95% CI)	APC 2 (95% CI)	APC 3 (95% CI)	AAPC (95% CI)
C73					
All women (*n* = 36,050)	1976; 2006	+4.61 (+1.90, +7.39)	+0.52 (+0.28, +0.77)	+5.83 (+4.56, +7.12)	+2.18 (+1.73, +2.64)
All men (*n* = 13,078)	2005	+0.35 (+0.09, +0.61)	+5.48 (+3.92, +7.06)	-	+1.55 (+1.15, +1.96)

## References

[1] Schneider A.B. (1990). Radiation-induced thyroid tumors. Endocrinol. Metab. Clin. N. Am..

[2] Quimby E.H., Werner S.C. (1949). Late Radiation Effects in Roentgen Therapy for Hyperthyroidism. JAMA.

[3] Duffy B.J., Fitzgerald P.J. (1950). Thyroid cancer in childhood and adolescence; a report on 28 cases. Cancer.

[4] Hallquist A., Hardell L., Löfroth P.O. (1993). External radiotherapy prior to thyroid cancer: A case-control study. Int. J. Radiat. Oncol. Biol. Phys..

[5] Prentice R.L., Kato H., Yoshimoto K., Mason M. (1982). Radiation exposure and thyroid cancer incidence among Hiroshima and Nagasaki residents. Natl. Cancer Inst. Monogr..

[6] Tsuda T., Tokinobu A., Yamamoto E., Suzuki E. (2016). Thyroid Cancer Detection by Ultrasound Among Residents Ages 18 Years and Younger in Fukushima, Japan: 2011 to 2014. Epidemiology.

[7] Zablotska L., Nadyrov E., Rozhko A., Gong Z., Polyanskaya O., McConnell R., O’Kane P., Brenner A., Little M.P., Ostroumova E. (2015). Analysis of thyroid malignant pathological findings identified during three rounds of screening (1997–2008) of a Belarusian cohort of children and adolescents exposed to radioiodines after the Chernobyl accident. Cancer.

[8] Tondel M., Hjalmarsson P., Hardell L., Carlsson G., Axelson O. (2004). Increase of regional total cancer incidence in north Sweden due to the Chernobyl accident?. J. Epidemiol. Community Health.

[9] Hallquist A., Hardell L., Degerman A., Boquist L. (1994). Thyroid cancer: Reproductive factors, previous diseases, drug intake, family history and diet. A case-control study. Eur. J. Cancer Prev..

[10] Cao Y., Wang Z., Gu J., Hu F., Qi Y., Yin Q., Sun Q., Li G., Quan B. (2015). Reproductive Factors but Not Hormonal Factors Associated with Thyroid Cancer Risk: A Systematic Review and Meta-Analysis. Biomed Res. Int..

[11] Carlberg M., Hedendahl L., Ahonen M., Koppel T., Hardell L. (2016). Increasing incidence of thyroid cancer in the Nordic countries with main focus on Swedish data. BMC Cancer.

[12] Vigneri R., Malandrino P., Vigneri P. (2015). The changing epidemiology of thyroid cancer: Why is incidence increasing?. Curr. Opin. Oncol..

[13] Adas M., Adas G., Koc B., Ozulker F. (2015). Incidental thyroid lesions on FDG-PET/CT: A prevalence study and proposition of management. Minerva Endocrinol..

[14] Lauer O., Frei P., Gosselin M.-C., Joseph W., Röösli M., Fröhlich J. (2013). Combining near- and far-field exposure for an organ-specific and whole-body RF-EMF proxy for epidemiological research: A reference case. Bioelectromagnetics.

[15] Luo J., Deziel N.C., Huang H., Chen Y., Ni X., Ma S., Udelsman R., Zhang Y. (2019). Cell phone use and risk of thyroid cancer: A population-based case-control study in Connecticut. Ann. Epidemiol..

[16] Luo J., Li H., Deziel N.C., Huang H., Zhao N., Ma S., Ni X., Udelsman R., Zhang Y. (2020). Genetic susceptibility may modify the association between cell phone use and thyroid cancer: A population-based case-control study in Connecticut. Environ. Res..

[17] National Toxicology Program (2018). NTP Technical Report on the Toxicology and Carcinogenesis Studies in B6C3F1/N Mice Exposed to Whole-Body Radio Frequency Radiation at a Frequency (1900 MHz) and Modulations (GSM and CDMA) Used by Cell Phones. https://ntp.niehs.nih.gov/ntp/about_ntp/trpanel/2018/march/tr596peerdraft.pdf.

[18] National Toxicology Program NTP Technical Report on the Toxicology and Carcinogenesis Studies in Hsd:Sprague Dawley sd Rats Exposed to Whole-Body Radio Frequency Radiation at a Frequency (900 MHz) and Modulations (GSM and CDMA) Used by Cell Phones. https://ntp.niehs.nih.gov/ntp/about_ntp/trpanel/2018/march/tr595peerdraft.pdf.

[19] Hardell L., Carlberg M. (2019). Comments on the US National Toxicology Program technical reports on toxicology and carcinogenesis study in rats exposed to whole-body radiofrequency radiation at 900 MHz and in mice exposed to whole-body radiofrequency radiation at 1900 MHz. Int. J. Oncol..

[20] Kim H.J., Fay M.P., Feuer E.J., Midthune D.N. (2000). Permutation tests for joinpoint regression with applications to cancer rates. Stat. Med..

[21] Caumo S., Vicente A., Custódio D., Alves C., Vasconcellos P. (2018). Organic compounds in particulate and gaseous phase collected in the neighbourhood of an industrial complex in São Paulo (Brazil). Air Qual. Atmos. Health.

[22] Shore R.E., Hempelmann L.H., Woodward A.D., Upton A.C., Albert R.E., Burns F.J., Shore R.E. (1986). Carcinogenic effects of radiation on the human thyroid gland. Radiation Carcinogenesis.

[23] Rahib L., Smith B.D., Aizenberg R., Rosenzweig A.B., Fleshman J.M., Matrisian L.M. (2014). Projecting cancer incidence and deaths to 2030: The unexpected burden of thyroid, liver, and pancreas cancers in the United States. Cancer Res..

[24] Sanabria A., Kowalski L.P., Shah J.P., Nixon I.J., Angelos P., Williams M.D., Rinaldo A., Ferlito A. (2018). Growing incidence of thyroid carcinoma in recent years: Factors underlying overdiagnosis. Head Neck.

[25] Vaccarella S., Dal Maso L., Laversanne M., Bray F., Plummer M., Franceschi S. (2015). The Impact of Diagnostic Changes on the Rise in Thyroid Cancer Incidence: A Population-Based Study in Selected High-Resource Countries. Thyroid.

[26] Socialstyrelsen [The National Board of Health and Welfare] Statistics on Cancer Incidence 2018. https://www.socialstyrelsen.se/globalassets/sharepoint-dokument/artikelkatalog/statistik/2019-12-6525.pdf.

[27] Wingren G., Hallquist A., Hardell L. (1997). Diagnostic X-ray exposure and female papillary thyroid cancer: A pooled analysis of two Swedish studies. Eur. J. Cancer Prev..

[28] Braganza M.Z., Potischman N., Park Y., Thompson F.E., Hollenbeck A.R., Kitahara C.M. (2015). Adolescent and mid-life diet and subsequent risk of thyroid cancer in the NIH-AARP Diet and Health Study. Int. J. Cancer.

